# Functional analysis of CfSnf1 in the development and pathogenicity of anthracnose fungus *Colletotrichum fructicola* on tea-oil tree

**DOI:** 10.1186/s12863-019-0796-y

**Published:** 2019-12-05

**Authors:** Shengpei Zhang, Yuan Guo, Sizheng Li, Guoying Zhou, Junang Liu, Jianping Xu, He Li

**Affiliations:** 1 College of Forestry, Central South University of Forestry and Technology and Key Laboratory of National Forestry and Grassland Administration on Control of Artificial Forest Diseases and Pests in South China, Changsha, China; 2 Hunan Provincial Key Laboratory for Control of Forest Diseases and Pests, Key Laboratory for Non-wood Forest Cultivation and Conservation of Ministry of Education, Changsha, China; 3 Department of Biology, McMaster University, Hamilton, Ontario Canada

**Keywords:** Conidiation, Appressorium formation, Pathogenicity, *C. fructicola*

## Abstract

**Background:**

Tea-oil tree (*Camellia oleifera*) is a unique edible-oil tree in China, and anthracnose occurs in wherever it is cultivated, causing great economic losses each year. We have previously identified the Ascomycete fungus *Colletotrichum fructicola* as the major pathogen of anthracnose in *Ca.* *oleifera*. The purpose of this study was to characterize the biological function of Snf1 protein, a key component of the AMPK (AMP-activated protein kinase) pathway, for the molecular pathogenic-mechanisms of *C. fructicola*.

**Results:**

We characterized CfSnf1 as the homolog of *Saccharomyces cerevisiae* Snf1. Targeted *CfSNF1* gene deletion revealed that CfSnf1 is involved in the utilization of specific carbon sources, conidiation, and stress responses. We further found that the Δ*CfSnf1* mutant was not pathogenic to *Ca.* *oleifera*, resulting from its defect in appressorium formation. In addition, we provided evidence showing crosstalk between the AMPK and the cAMP/PKA pathways for the first time in filamentous fungi.

**Conclusion:**

This study indicate that CfSnf1 is a critical factor in the development and pathogenicity of *C. fructicola* and, therefore, a potential fungicide target for anthracnose control.

## Background

Tea-oil tree (*Camellia oleifera*) is a commercial shrub native to China and has been widely cultivated throughout southern China for more than 2000 years with abundant edible oil in its seeds [1]. Owing to its lower cholesterol concentrations and the ability to decrease lipid concentration and prevent hypertension and arteriosclerosis, tea oil is considered as an excellent source of oil for human health, similar to that of olive oil [2, 3]. In China, Tea-oil tree covers more than 30,000 km^2^ and produces 250,000 tons of edible oil each year, however, it is still unable to meet the consumer demand for tea oil. One of the major limiting factors is the serious diseases occurred in Tea-oil tree.

Anthracnose is the most devastating disease in Tea-oil tree and happens in wherever it is cultivated [4]. The buds, fruits, and leaves of Tea-oil tree all are susceptible to the disease, causing the wilt or even fall of the plant tissues. Anthracnose typically results in 10%~ 30% reduction of tea oil each year, and the severely affected areas often experience more than 50% of tea oil losses. Our previous studies demonstrated that there are at least five pathogens of anthracnose in Tea-oil tree, namely: *Colletotrichum fructicola*, *Colletotrichum siamense*, *Colletotrichum gloeosporioides*, *Colletotrichum camelliae*, and *Colletotrichum karstii*. Among them, *C. fructicola* showed the widest distribution and highest pathogenicity, acting as the major pathogen [4, 5].

*C. fructicola* was first identified in coffee berries in northern Thailand in 2009 and has been found to cause diseases in more than 50 plants, including apple, pear, strawberry *etc*; and was distributed broadly on five continents [6–9]. However, despite its ecological and economic importance, very little is known about its molecular pathogenesis. The recent transcriptomic analysis showed that there are many putative pathogenic-related genes in *C. fructicola*, and most of them are significantly up-regulated in early infectious stages [6, 10]. However, the functions of these putative pathogenic-related genes remains confirmed.

*Colletotrichum* is among the top 10 fungal pathogens of plants, with infections primarily start with conidia, the asexual spores [11]. Briefly, conidia attach onto the host surface and germinate to produce germ tubes, the end of which will develop into specialized infection structures named appressoria. The enormous turgor pressure of the appressoria help the fungi to penetrate and colonize host tissues, causing disease lesions on plants. Each infected tissue may produce thousands of conidia that when released, can initiate a new disease cycle on new plant tissues [12, 13]. When first attaching on the host surface, the conidia probably lack nutrients and energy, thus changes in metabolic processes for pathogens are needed in order to grow and infect. There are at least three signaling pathways involved in nutrient metabolism in organisms, namely AMPK (AMP-activated protein kinase), cAMP-PKA (cAMP dependent protein kinase A), and TOR (target of rapamycin) [14, 15]. Among them, AMPK and cAMP-PKA are mainly involved in the carbon metabolisms and TOR responses to the nitrogen/ amino acid metabolisms. Previous studies have systematically demonstrated that the cAMP-PKA and TOR pathways regulate pathogenicity through host surface sensing in filamentous fungi [16–18]. However, little is known about the roles of AMPK pathway in filamentous fungi.

AMPK pathway is also called Snf1 (sucrose non-fermenting 1) pathway in *Saccharomyces cerevisiae*, which is a heterotrimeric complex composed of the catalytic α subunit Snf1, a regulatory β subunit (one of the Gal83, Sip1 and Sip2) and the γ subunit Snf4 [19]. The serine/threonine protein kinase Snf1 is at the center of the heterotrimeric complex and is required for the adaptation of yeast cells to glucose limitation [20]. In high glucose concentrations Snf1 is inactivated. In contrast, when glucose is limited, the threonine residue of Snf1 is phosphorylated and activated [21]. The active Snf1 regulates the expression of more than 400 genes involved in adaptation to nutrient stress [22]. Besides the roles in carbon metabolism, more and more studies have revealed that Snf1 plays important roles in autophagy, aging, biofilm formation, mitochondrial homeostasis and the response to various environmental stresses [20, 23–25]. To date, Snf1 homologs have been identified in a small number of filamentous fungi and found to play critical roles in the pathogenicity of *Magnaporthe oryzae*, *Verticillium dahliae*, and *Cochliobolus carbonum* [26–28]. Despite the importance, no information is available regarding the roles of such proteins in forest fungal pathogens including *C. fructicola*. Here, we identified the protein kinase CfSnf1 from *C. fructicola* and characterized its functions.

## Results

### Identification and phylogenetic analysis of CfSnf1 in *C. fructicola*

**Fig. 1:**
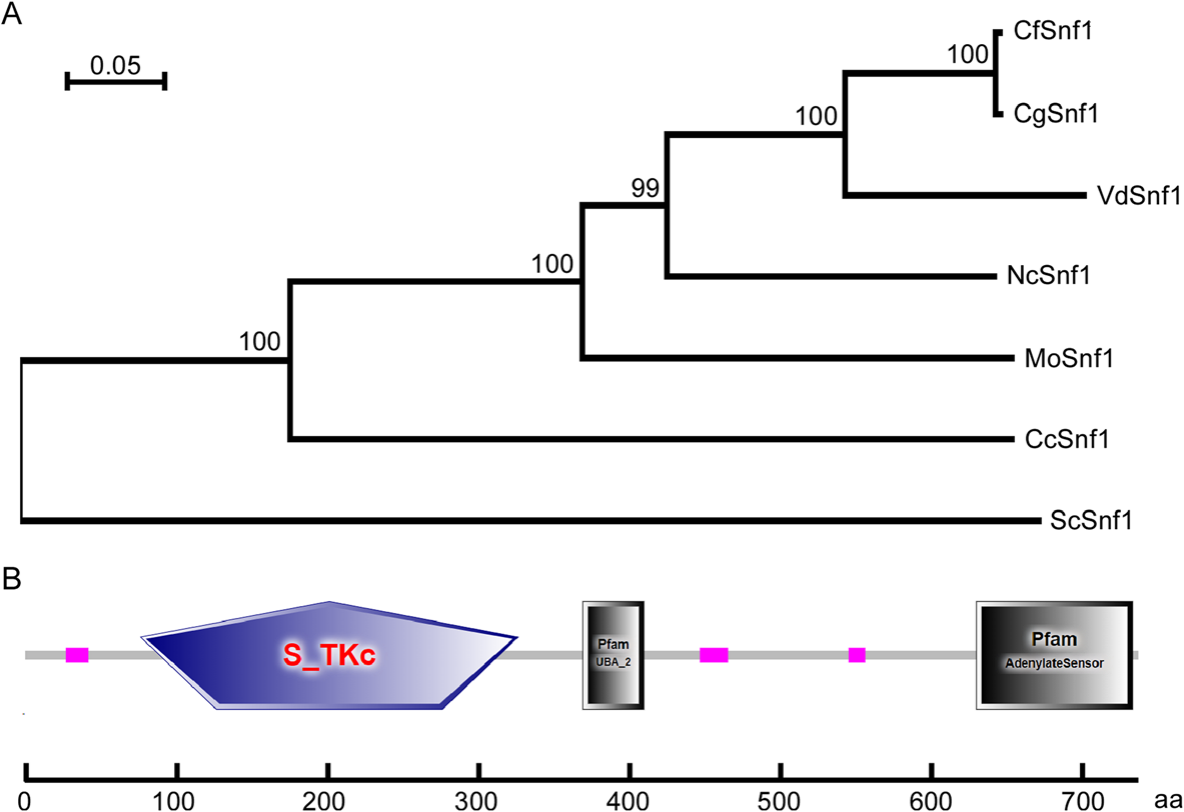
Phylogenetic analysis and domain prediction of CfSnf1. (**a**) The Snf1 proteins from diverse fungi were aligned using the CLUSTAL_W and the neighbour-joining tree was constructed by MEGA 5.05 with 1000 bootstrap replicates. The organisms and GenBank accession numbers are shown as follows: CfSnf1 (*C. fructicola*, MN094751); CgSnf1 (*C. gloeosporioides*, EQB56372.1); VdSnf1 (*V. dahliae*, RXG48310.1); NcSnf1 (*N. crassa*, XP_958665.3); MoSnf1 (*M. oryzae*, XP_003718181.1); CcSnf1 (*C. carbonum*, AF159253); ScSnf1 (*S. cerevisiae*, NP_010765.3). (**b**) The domains of CfSnf1 were predicted. The purple pentagon indicates the S_TKc (Serine/Threonine protein kinases, catalytic) domain, the gray quadrilaterals represent the UBA (ubiquitin-associated) domain and the Adenylate Sensor domain, and pink boxes refer to three low complexity regions

Using *S. cerevisiae* Snf1 sequence as the trace, we acquired its single homolog in *C. fructicola* genome database by a BLAST_P search. Then, we submitted its sequence to NCBI database (https://www.ncbi.nlm.nih.gov/) (GenBank accession number MN094751) and named it CfSnf1. CfSnf1 was predicted to encode 741 amino acids and phylogenetic analysis revealed that CfSnf1 shows sequence conservation among other fungi Snf1 proteins; CfSnf1 shows higher amino acid sequence homology with that of *C. gloeosporioides* (96% identify and 96% similarity) and lesser homology with *S. cerevisiae* Snf1 (still 43% identify and 56% similarity) (Fig. 1a). This result indicates that Snf1 proteins are well conserved in fungi.

The domain prediction using the SMART website (http://smart.embl-heidelberg.de/) suggested that CfSnf1 contains a S_TKc (Serine/Threonine protein kinases, catalytic) domain, a UBA (ubiquitin-associated) domain, an Adenylate Sensor domain, and three low complexity regions (Fig. 1b).

### Targeted deletion of *CfSNF1* gene in *C. fructicola*

To characterize the functions of CfSnf1, a *CfSNF1* gene-deletion construct was generated according to the homologous recombination principle by replacing the coding region with a hygromycin-resistance cassette (*HPH*) (Additional file 1: Figure S1a). Putative transformants were screened on hygromycin media, then verified by PCR amplification, and we thus obtained two deletion mutants Δ*CfSnf1*#3 and Δ*CfSnf1*#5 (Additional file 1: Figure S1b). Since these two mutants showed the same biological phenotypes, we selected Δ*CfSnf1*#3 for further analysis. To complement the mutant strain, the genomic DNA sequence of *CfSNF1* containing a 1.5-kb promoter was retransformed to the Δ*CfSnf1*#3 mutant and restored all of the mutant defects.

### CfSnf1 is involved in the utilization of specific carbon sources and aerial hyphae growth

**Fig. 2:**
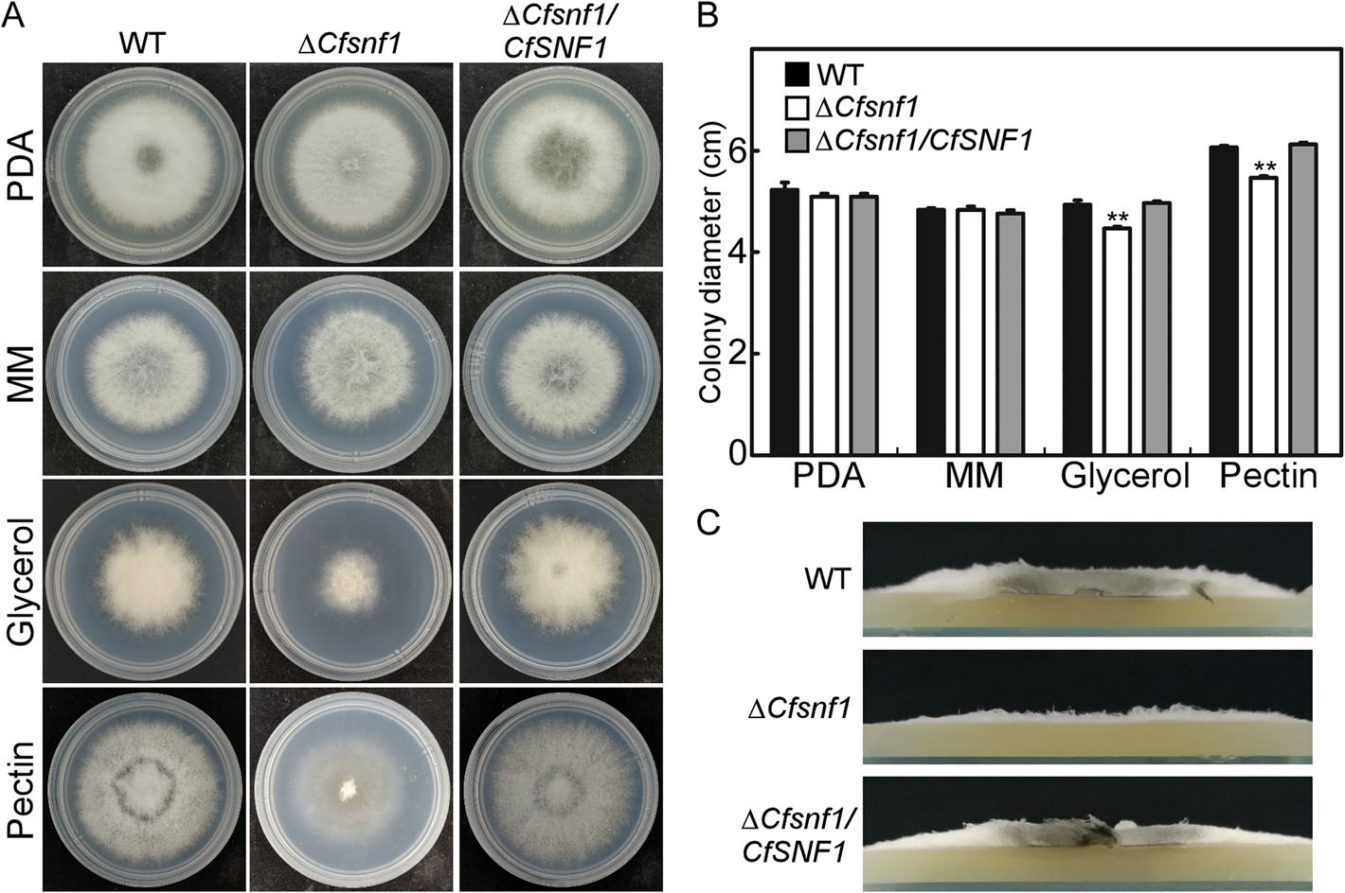
CfSnf1 is involved in the utilization of specific carbon sources and aerial hyphae growth. (**a**) Growth of WT, Δ*CfSnf1* mutant, and complemented strain Δ*CfSnf1*/*CfSNF1* on the PDA, MM and MM plates supplemented with 1% Glycerol or 1% Pectin as sole carbon source for 4 days at 28 °C. (**b**) Colony diameters were measured and statistically analyzed by Duncan analysis from three replicates. Asterisks indicate significant differences (*p < 0.01*). (**c**) Aerial hyphae growth is reduced in Δ*CfSnf1* mutant. Strains were cultured in PDA for 4 days and colony side views are shown from three replicates

Snf1 proteins have been demonstrated to be important for the utilization of specific carbon sources in *V. dahliae* and *M. oryzae* [26, 27]. To investigate the functions of CfSnf1 in utilization of carbon sources, Wide-Type (WT), Δ*CfSnf1* mutant, and complemented strain Δ*CfSnf1*/*CfSNF1* were cultured in the plates of PDA (Potato Dextrose Agar), MM (Minimal Medium), and MM supplemented with 1% Glycerol or 1% Pectin as sole carbon source for 4 days. All the strains showed similar growth levels on PDA and MM, while Δ*CfSnf1* mutant showed significantly reduced growth rates to WT and Δ*CfSnf1*/*CfSNF1* on MM supplemented with Glycerol or Pectin (Fig. 2a and b). Meanwhile, we also found that Δ*CfSnf1* mutant exhibit a flat colony compared with the fluffy colony of WT and Δ*CfSnf1*/*CfSNF1*, owing to its reduced aerial hyphal growth (Fig. 2a and c).

### CfSnf1 is important in asexual development

**Fig. 3:**
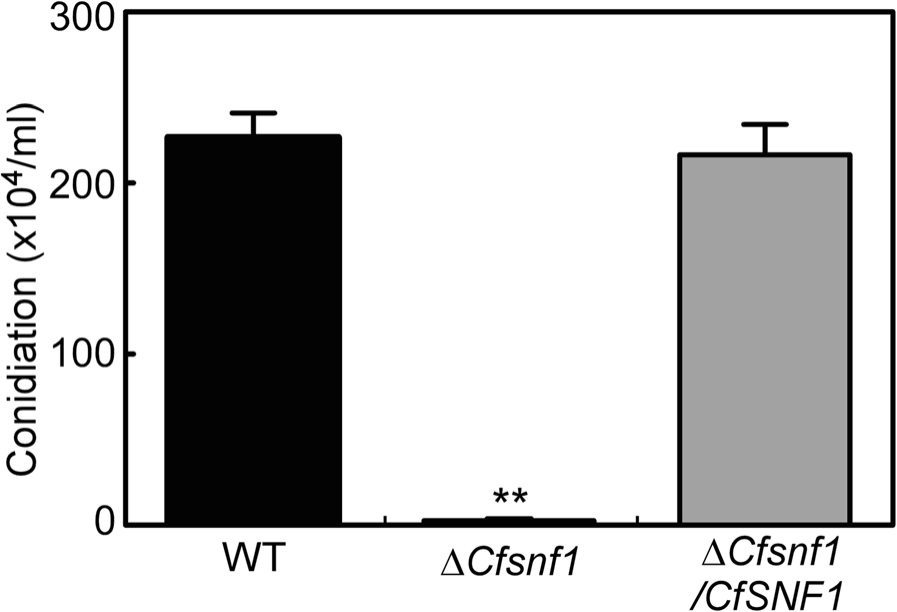
CfSnf1 is important in asexual development. The conidia produced by WT, Δ*CfSnf1* and Δ*CfSnf1*/*CfSNF1* cultured in PDB medium for 4 days were collected and quantified, then analyzed by Duncan analysis (*p < 0.01*) from three replicates. Three independent experiments yielded similar results

Like most fungal pathogens, asexual conidia are important for the disease cycle and infection in *Colletotrichum* [29, 30]. To examine the role of CfSnf1 in conidiation, WT, Δ*CfSnf1* mutant, and Δ*CfSnf1*/*CfSNF1* were cultured in liquid shaking PDB for 4 days. WT and Δ*CfSnf1*/*CfSNF1* showed more than 220 × 10^4^ conidia per milliliter, comparing with the less than 5 × 10^4^ conidia per milliliter for Δ*CfSnf1* mutant (Fig. 3). This result demonstrates that CfSnf1 is important in conidiation.

### CfSnf1 is essential for pathogenicity

**Fig. 4:**
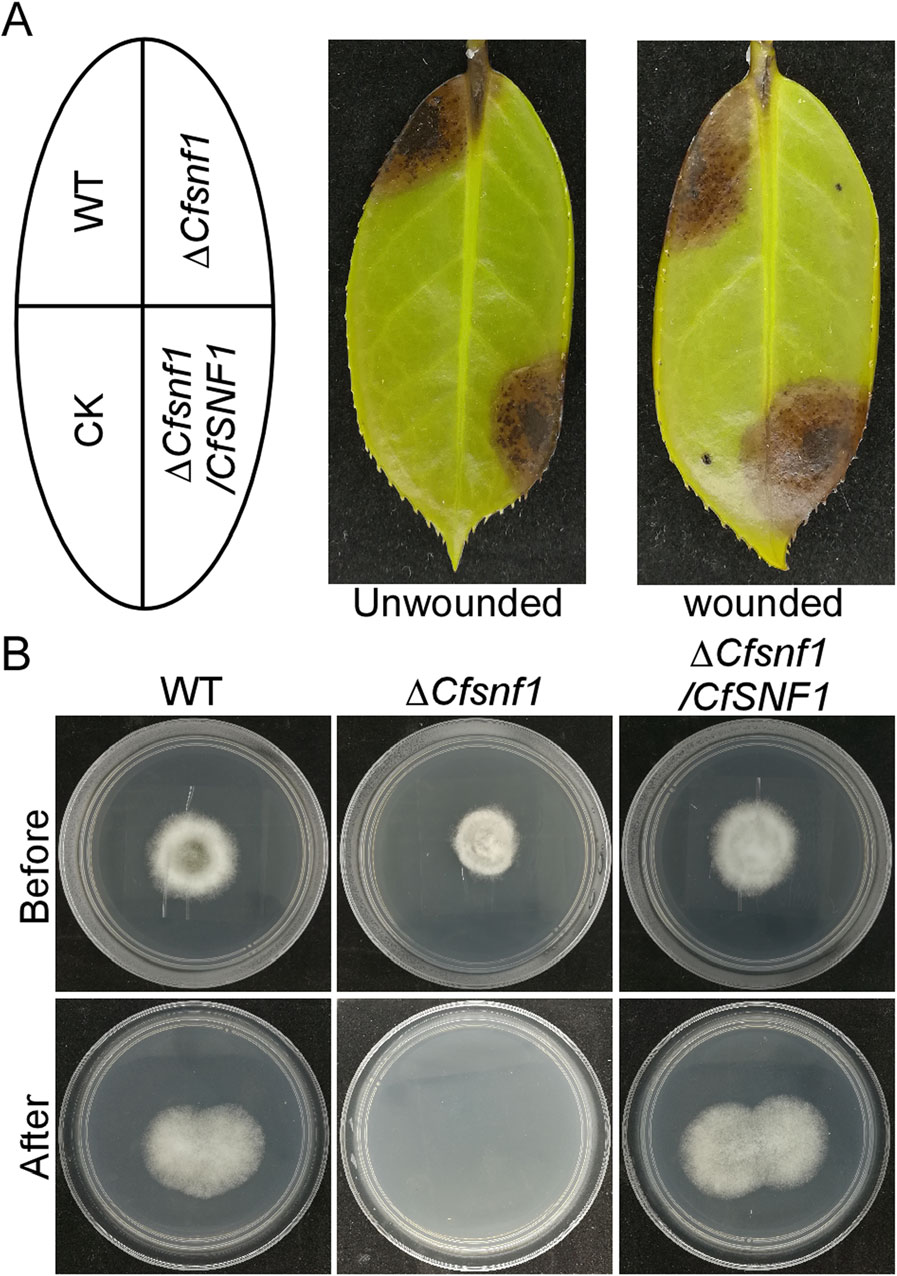
CfSnf1 is essential for pathogenicity. (**a**) Mycelial plugs of the WT, Δ*CfSnf1* and Δ*CfSnf1*/*CfSNF1* were inoculated onto unwounded or wounded *C. oleifera* leaves with three replicates, and photographed at 4 days post inoculation (dpi), CK: controls. (**b**) Colonies of the strains with three replicates were cultured on top of cellophane membranes placed on PDA plates (Before). After 3 days incubation, the cellophane membranes with fungal dishes were removed, and the plates were further cultured for 3 days and photographed (After)

As a plant-pathogenic fungus, we concentrated our interests on the role of pathogenicity for CfSnf1 in *C. fructicola*. Mycelial plugs of the WT, Δ*CfSnf1* mutant, and Δ*CfSnf1*/*CfSNF1* were inoculated onto the intact Ca. *oleifera* leaves. After 4 days incubation, Δ*CfSnf1* mutant caused no lesions, in contrast to the large and typical lesions produced by the WT and Δ*CfSnf1*/*CfSNF1* (Fig. 4a left panel). To examine whether CfSnf1 plays roles in infectious growth, we further carried out the pathogenicity assay on wounded leaves. The result showed that Δ*CfSnf1* mutant still causes no lesions, compared with the typical lesions of WT and Δ*CfSnf1*/*CfSNF1* (Fig. 4a right panel). This result indicates that CfSnf1 is essential for pathogenicity.

To further analyze the pathogenicity defect of Δ*CfSnf1* mutant, we performed the cellophane penetration assay. Colonies of the strains were first cultured on top of cellophane membranes placed on PDA plates for 3 days incubation, then the cellophane membranes with fungal dishes were removed. After the further incubation for 3 days, we found Δ*CfSnf1* mutant could not penetrate the cellophane, compared with the mycelial growth of WT and Δ*CfSnf1*/*CfSNF1* on the plates (Fig. 4b). This result suggests that CfSnf1 is required for penetration.

### CfSnf1 is localized to cytoplasm

**Fig. 5:**
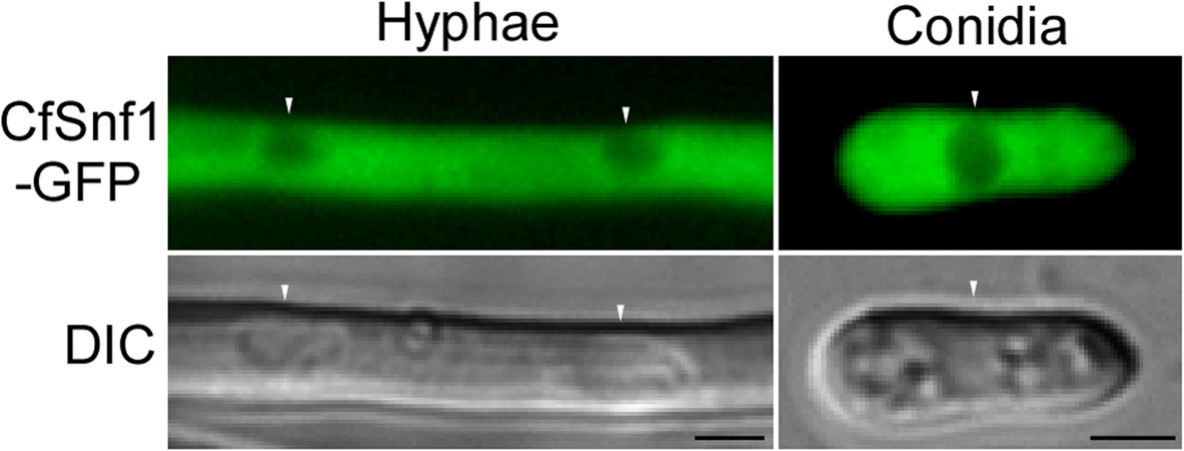
CfSnf1 is localized to cytoplasm. The localization pattern of CfSnf1 in the hyphae and conidia. Three independent biological experiments were carried out with three replicates each time. Arrows indicate vacuoles. DIC: differential interference contrast. Bar = 5 μm

To detect the subcellular localization of CfSnf1, we fused a green fluorescent protein (GFP) tag to the C-terminus of CfSnf1 and introduced it into the Δ*CfSnf1* mutant. Strong GFP signals were uniformly distributed throughout the cytoplasm, but not in vacuoles, both in hyphae and conidia (Fig. 5). This result suggests the expression and cytoplasm-localized pattern of CfSnf1 in hyphae and conidia.

### CfSnf1 is involved in response to osmotic stress

**Fig. 6:**
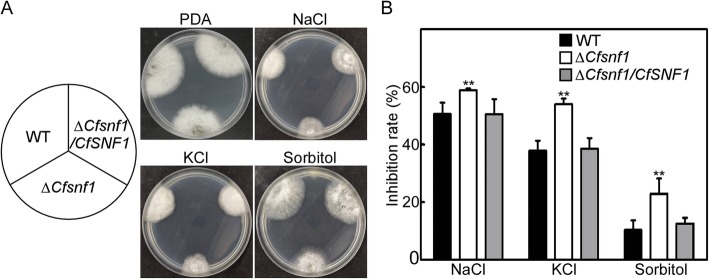
CfSnf1 is involved in response to osmotic stress. (**a**) The WT, Δ*CfSnf1* and Δ*CfSnf1*/*CfSNF1* strains were cultured onto PDA plates with different osmotic stresses (NaCl, KCl, and Sorbitol) at 28 °C for 4 days and photographed. (**b**) Statistical analysis of inhibition rates of the strains to osmotic stress. Three independent biological experiments were carried out with three replicates each time, and similar results were obtained among the biological replicates. Error bars represent SD of three replicates and asterisks represent significant differences (*p < 0.01*)

For normal growth and infection, fungi must undergo many types of stresses in nature, such as osmotic stress, oxidative stress and cell wall integrity stress etc. Studies in yeast concluded that ScSnf1 plays critical roles in response to various environmental stresses [20, 31, 32]. Here we investigated the roles of CfSnf1 in the response to environmental stresses. We first cultured the WT, Δ*CfSnf1* mutant, and Δ*CfSnf1*/*CfSNF1* on PDA plates supplemented with osmotic stress (1 M NaCl, 1 M KCl, and 1 M sorbitol) for 4 days. We found that Δ*CfSnf1* mutant showed significant higher inhibition rates than that of WT and Δ*CfSnf1*/*CfSNF1* in all three osmotic stresses (Fig. 6a and b). These results indicate that CfSnf1 is involved in response to osmotic stress. Then, we examined the sensitivity of the strains to two cell wall integrity inhibitors, Congo red (CR) and sodium dodecyl sulfate (SDS), and oxidative stress H_2_O_2_. Consistently, all the strains showed similar inhibition rates on CR, SDS and H_2_O_2_ plates (Additional file 2:Fig. S2).

### CfSnf1 is required for appressorium formation

**Fig. 7:**
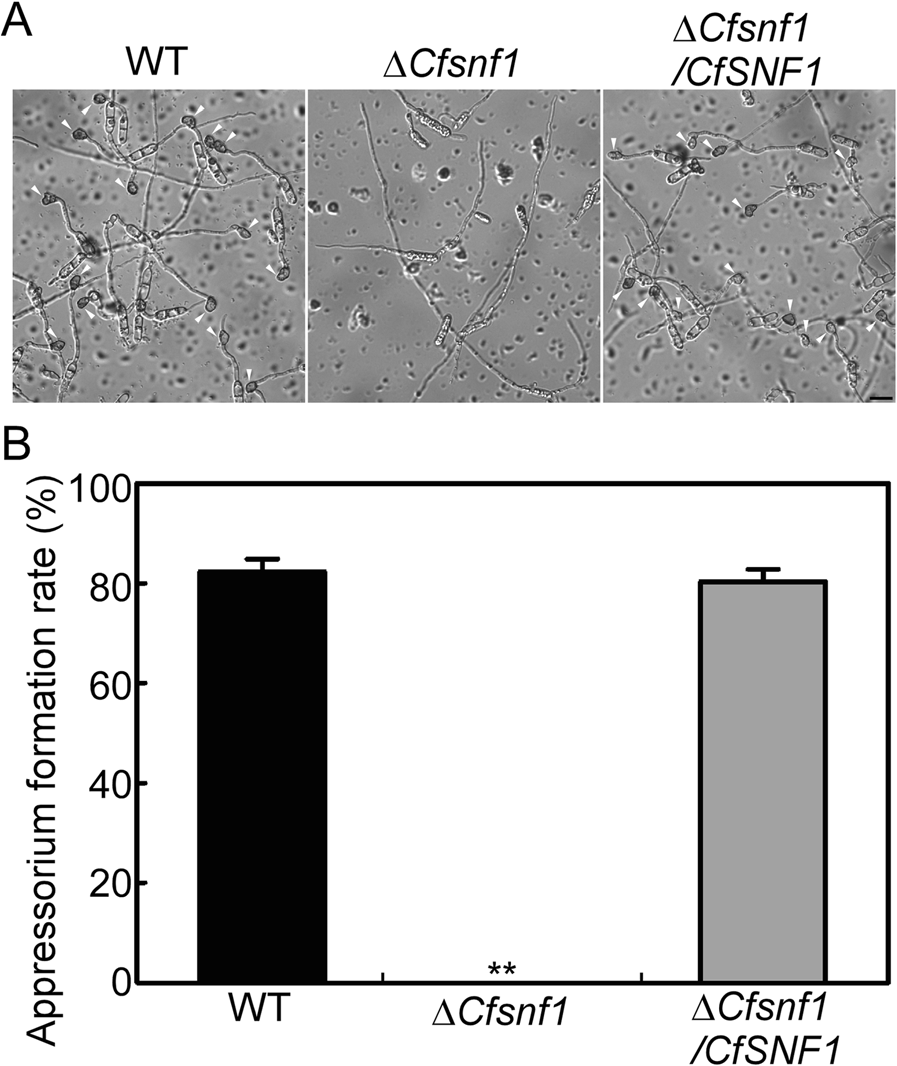
CfSnf1 is required for appressorium formation. (**a**) Conidia collected from the WT, Δ*CfSnf1* and Δ*CfSnf1*/*CfSNF1* strains were inoculated onto hydrophobic artificial surfaces at 24 h post inoculation (hpi) and photographed. Arrows indicate appressoria. (**b**) The percentage of appressorium formation was statistically analyzed. Error bars represent SD and asterisks indicate statistically significant differences (*p* < 0.01) from three replicates. Bar = 10 μm

Based on the fact that Δ*CfSnf1* mutant caused no lesions on both unwounded and wounded tea-oil tree leaves and its non-penetration on cellophane membranes, we wondered whether the mutant could not produce any functional appressorium. To further elucidate the mechanisms of the abolished pathogenicity in Δ*CfSnf1* mutant, the conidia of WT, Δ*CfSnf1* and Δ*CfSnf1*/*CfSNF1* were inoculated onto hydrophobic artificial surfaces to induce the appressorium formation. At 4 h post inoculation (hpi), all the strains exhibited more than 80% conidial germination rate and showed no significant difference among them. However, the germ tubes of Δ*CfSnf1* were elongated abnormally and no appressoria were developed at 24 hpi, in contrast with the normal germ tubes and more than 80% appressorium formation rate in WT and Δ*CfSnf1*/*CfSNF1* (Fig. 7a and b). This result indicates that CfSnf1 is required for appressorium formation.

### cAMP treatment partially restores the appressorium formation defect of Δ*CfSnf1* mutant

**Fig. 8:**
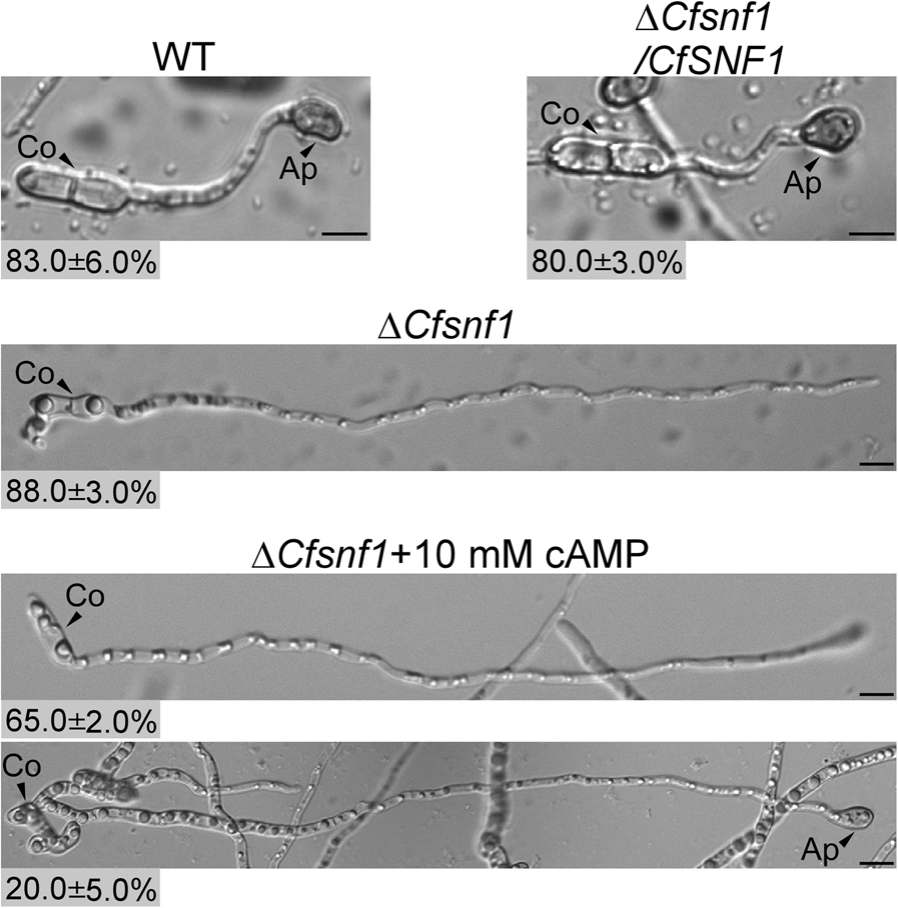
cAMP treatment partially restored the appressorium formation of Δ*CfSnf1* mutant. Appressorium formation rates were determined at 24 hpi from 100 spores per hydrophilic slide for WT, Δ*CfSnf1*, Δ*CfSnf1*/*CfSNF1* and Δ*CfSnf1* with 10 mM cAMP treatment, repeated in triplicate with three replicates each time. Co: Conidia; Ap: Appressorium. Bar = 10 μm

Studies in *M. oryzae*, which acts as the model for studying the mechanisms of fungal pathogenesis and host-microbe interactions, have revealed that the loss of cAMP/PKA signaling pathway abolishes appressorium formation and appressorium formation can be rescued in strains carrying deletions upstream of cAMP/PKA by cAMP treatment [31, 33, 34]. Therefore, we performed cAMP treatment for Δ*CfSnf1*, when inducing appressorium formation. The result showed that 88% of Δ*CfSnf1* conidia germinated but do not formed appressoria and the rest conidia were not germinated, however, about 20% of Δ*CfSnf1* conidia formed appressoria when cAMP was added (Fig. 8). This result reveals that cAMP treatment could partially restore the appressorium formation defect of Δ*CfSnf1* mutant.

## Discussion

In the present study, we characterized CfSnf1 as the homolog of *S. cerevisiae* Snf1 in *C. fructicola*. We found that CfSnf1 plays critical roles in the utilization of specific carbon sources, conidiation, appressorium formation, stress responses and pathogenicity in *C. fructicola*.

Targeted *CfSNF1* gene deletion mutant Δ*CfSnf1* showed similar mycelial growth rate as the WT in PDA and MM plates, but showed decreased growth rate compared to WT on MM with Glycerol or Pectin as sole carbon source. Considering the fact that PDA and MM medium use glucose for carbon source and the yeast Snf1 is inactivated in high glucose concentrations and activated in low glucose [21], we speculate that CfSnf1 also mainly functions in low glucose condition in *C. fructicola*. Moreover, the homologs of Snf1 in *V. dahliae* and *C. carbonum* are also required for the utilization of specific carbon sources [26, 27], which is consistent with our study. These findings indicate that Snf1 proteins shared conserved mechanisms during carbon utilization.

Asexual conidia play critical roles in the disease cycle and infection of *Colletotrichum* [29, 30]. The Δ*CfSnf1* mutant produced dramatically decreased conidiation, which is consistent with the studies in *M. oryzae* [28, 35]. However, the Δ*CcSnf1* mutant showed normal conidiation in *C. carbonum* and the Δ*VdSnf1* mutant even produced more conidia in *V. dahliae* [26, 27]. These differences in conidiation by *SNF1* mutants of the fungal pathogens reflect distinct functions of Snf1 proteins during asexual development in different fungal species.

The studies in yeast revealed the roles of Snf1 in response to various environmental stresses through the activation of downstream proteins [20, 36]. Our study demonstrated that CfSnf1 is involved in response to osmotic stress but plays no role in the response to cell wall integrity stress and oxidative stress in *C. fructicola*. Such differences probably contribute to the specificity of CfSnf1 in stress responses. Furthermore, since fungi must undergo many types of stresses in nature for normal infection, the function of CfSnf1 in osmotic stress response might foretell its role in pathogenicity.

The Δ*CfSnf1* mutant were non-pathogenic on intact Tea-oil leaves, indicating the penetrating defect of the mutant and its non-penetration on cellophane membranes confirmed this. Furthermore, the lack of pathogenicity on wounded Tea-oil leaves also revealed the host-colonizing defect of Δ*CfSnf1* mutant. We reasoned that the abolished pathogenicity of Δ*CfSnf1* mutant was directly due to the defect in appressorium formation, which is essential for the penetration and host-colonization of *Colletotrichum* [29, 30]. In addition, the defect of appressorium formation in Δ*CfSnf1* mutant was partially restored by cAMP, which is a positive determinant in cAMP/PKA pathway [31]. Thus, we predict a relationship between Snf1 and cAMP/PKA pathway in *C. fructicola*, which has also been reported but not clearly clarified in yeast and mammalian cells [21, 37]. Though the related mechanism is unknown and needs to be further investigated, at least, we establish the first crosstalk between Snf1 and cAMP/PKA pathway in filamentous fungi.

## Conclusion

This study concluded that CfSnf1 is involved in utilization of specific carbon sources, conidiation, and stress responses in *C. fructicola*. CfSnf1 also functions as a key regulator for appressorium formation that are crucial for the pathogenicity of the fungus.

## Methods

### Strains and culture conditions

*C. fructicola* CFLH16 was used as the wild-type strain. All strains were cultured on PDA (200 g peeled potato, 20 g dextrose, and 15 g agar in 1 L ddH_2_O) plates at 28 °C in the darkness, unless indicated otherwise. The strains were cultured in liquid PDA medium in darkness shaking at 28 °C for 2 days and collected for the extraction of genomic DNA.

### Phylogenetic tree construction and domain prediction

The Snf1 proteins of *C. fructicola*, *C. gloeosporioides*, *V. dahliae*, *Neurospora crassa*, *M. oryzae*, *C. carbonum*, and *S. cerevisiae* were acquired from the NCBI database (https://www.ncbi.nlm.nih.gov/). The phylogenetic tree was constructed by MEGA 5.05 programs using neighbor-joining method with 1000 bootstrap replicates. The domain of CfSnf1 was predicted by the SMART website (http://smart.embl-heidelberg.de/).

### Gene deletion and complementation assays

*CfSNF1* targeted gene deletion was performed by one-step replacement strategy [38]. Two about 1.0-kb DNA fragments flanking the *CfSNF1* gene and 1.4-kb *HPH* gene were amplified using primes (Additional file 3: Table. S1), then the two flanking sequences were ligated to the flanks of *HPH*, respectively, by overlap PCR. The PCR products were further cloned into the pMD19-T vector. After sequencing, the 3.4-kb fragments, which contain the flanking sequences and hygromycin cassette, were amplified and transformed into the protoplasts of wild-type strain. The transformants were selected by hygromycin and screened by PCR. For complementation assays, approximately 1.5-kb native promoter and the full-length of *CfSNF1* were amplified and ligated to the pYF11 vector (bleomycin resistance). After sequencing, the fused-pYF11 plasmids were transformed into the protoplasts of Δ*CfSnf1* mutant for complementation.

### Growth assays on different carbon sources

The strains were cultured on PDA, MM (6 g NaNO_3_, 0.52 g KCl, 0.152 g MgSO_4_·7H_2_O, 1.52 g KH_2_PO_4_, 0.01 g VB1, 1 ml 1000 × trace elements, 10 g Glucose, and 15 g agar in 1 L ddH_2_O), and MM with the Glucose was substituted by 1% Glycerol or 1% Pectin. After 4 days incubation, the colony diameters were measured and statistically analyzed.

### Stress response assays

The strains were cultured on PDA and PDA added with different stresses, including osmotic stress (1 M NaCl, 1 M KCl, and 1 M Sorbitol), cell wall integrity stress (0.01% SDS and 400 μg/ml CR), and oxidative stress (10 mM H_2_O_2_). After 4 days incubation, the colony diameters were measured and the inhibition rates were statistically analyzed.

### Conidiation and appressorium formation assays

For conidiation, the strains were cultured in liquid PDB (Potato Dextrose Broth) for 4 days, then filtered with three layers of lens paper and the conidia were collected and statistically analyzed. For appressorium formation, the collected conidia were resuspended to a concentration of 1 × 10^5^ spores/mL and inoculated onto hydrophobic artificial surfaces for germination and appressorium formation. For cAMP treatment, the conidial suspensions of Δ*CfSnf1* mutant were added with 10 mM cAMP and then were induced for appressorium formation.

### Pathogenicity and penetration assays

For pathogenicity assay, the mycelial plugs of the strains were inoculated onto the leaf margin of the detached Tea-oil tree leaves. The inoculated leaves were kept in a humidity plate with a 12 h light and 12 h dark cycle for 4 days and then photographed. The penetration assay on cellophane membranes was performed as previously described [39, 40] with minor modifications, colonies of the strains were first cultured on top of cellophane membranes placed on PDA plates. After 3 days incubation, the cellophane membranes with fungal dishes were removed, and the plates were further cultured for 3 days.

### Localization observation

The green fluorescent protein (GFP) tag was fused to the C-terminus of CfSnf1 and transformed into the Δ*CfSnf1* mutant. The fluorescence of the hyphae and conidia was observed under microscope.

## Supplementary information

**Additional files 1: Figure S1.** Generation of the *CfSNF1* gene deletion mutants in *C. fructicola*. (A) Strategy for the construction of gene replacement fragment. ~ 1.0-kb left fragment (LF) and right fragment (RF) flanking the *CfSNF1* gene and 1.4-kb *HPH* gene from pCX62 vector were amplified, then the LF and RF were ligated to the flanks of *HPH*, respectively, by overlap PCR. The PCR products were further cloned into the pMD19-T vector and obtained the vector of pMD-*CfSNF1*-KO. After sequencing, the 3.4-kb fragments, which contain the LF, RF and *HPH*, were amplified for gene replacement. (B) Schematic illustration for deletion strategy of *CfSNF1* gene*.* (C) Validation of the gene deletion mutants by PCR amplified with primers 1 (NBF/NBR) and primers 2 (BWF/HPHR). M: marker; +: positive control; −: negative control; #3 and #5: mutants.

**Additional files 2: Figure S2.** CfSnf1 is not involved in the tolerance of cell wall stress and oxidative stress. (A) The WT, Δ*CfSnf1* and Δ*CfSnf1*/*CfSNF1* strains were cultured onto PDA plates with different cell wall stresses (CR and SDS) and oxidative stress (H_2_O_2_) at 28 °C for 4 days and photographed. (B) Statistical analysis of inhibition rates of the strains to cell wall stresses and oxidative stress. Error bars represent SD of three replicates.

**Additional files 3: Table S1.** Primers used in this study.

## Data Availability

All data generated or analyzed during this study are included in this published article and its supplementary additional files.

## Abbreviations

CR: Congo red
PCR: Polymerase chain reaction
SDS: Sodium dodecyl sulfate
