# Supplementary figures and images for: Functional analysis of CfSnf1 in the development and pathogenicity of anthracnose fungus Colletotrichum fructicola on tea-oil tree
Source: BMC Genet. 2019 Dec 5;20:94. doi: 10.1186/s12863-019-0796-y (PMC6896739; doi:10.1186/s12863-019-0796-y)

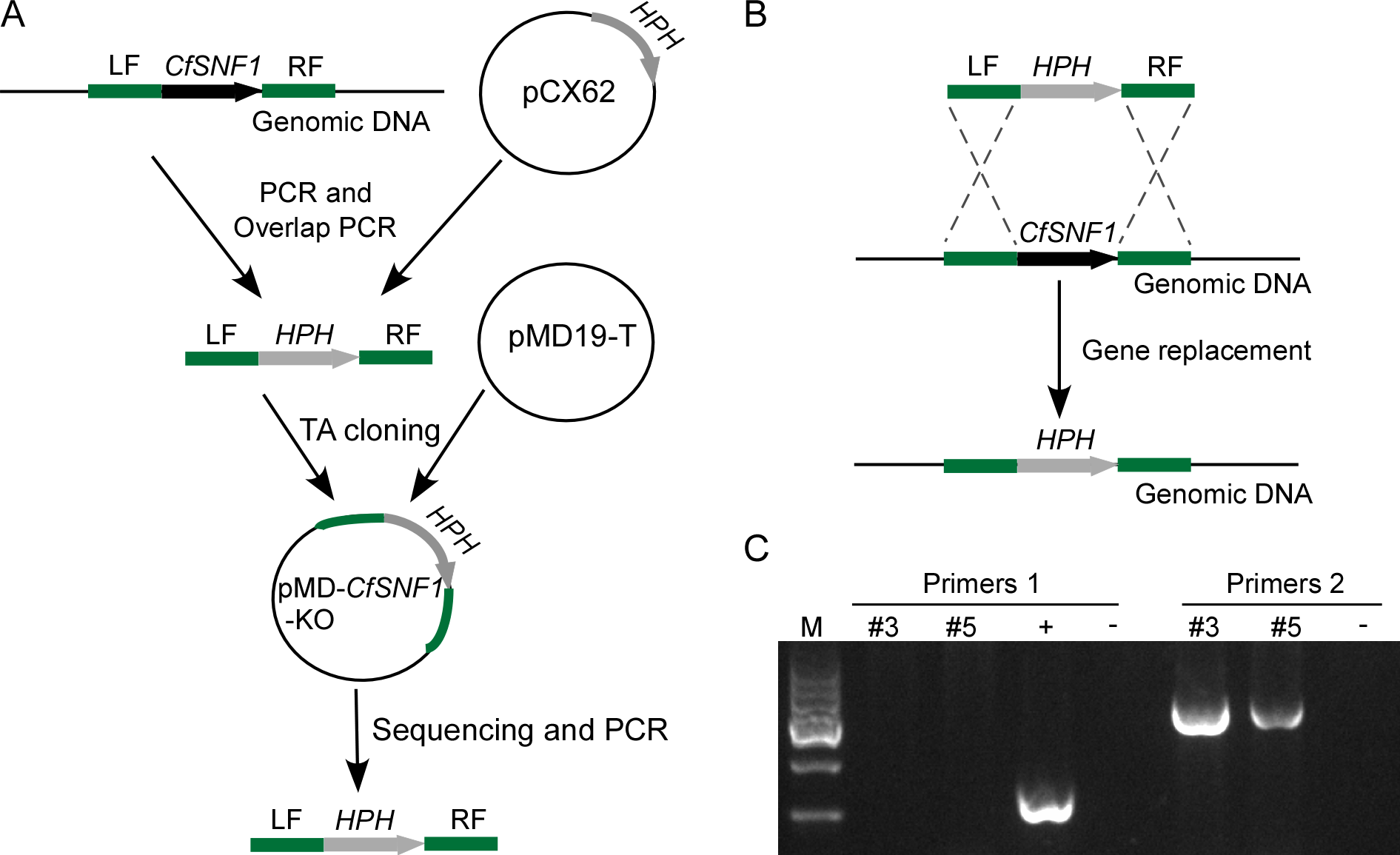

Supplement: Supplementary file 1 — Additional files 1: Figure S1. Generation of the CfSNF1 gene deletion mutants in C. fructicola. (A) Strategy for the construction of gene replacement fragment. ~ 1.0-kb left fragment (LF) and right fragment (RF) flanking the CfSNF1 gene and 1.4-kb HPH gene from pCX62 vector were amplified, then the LF and RF were ligated to the flanks of HPH, respectively, by overlap PCR. The PCR products were further cloned into the pMD19-T vector and obtained the vector of pMD-CfSNF1-KO. After sequencing, the 3.4-kb fragments, which contain the LF, RF and HPH, were amplified for gene replacement. (B) Schematic illustration for deletion strategy of CfSNF1 gene. (C) Validation of the gene deletion mutants by PCR amplified with primers 1 (NBF/NBR) and primers 2 (BWF/HPHR). M: marker; +: positive control; −: negative control; #3 and #5: mutants. [file 12863_2019_796_MOESM1_ESM.tif]

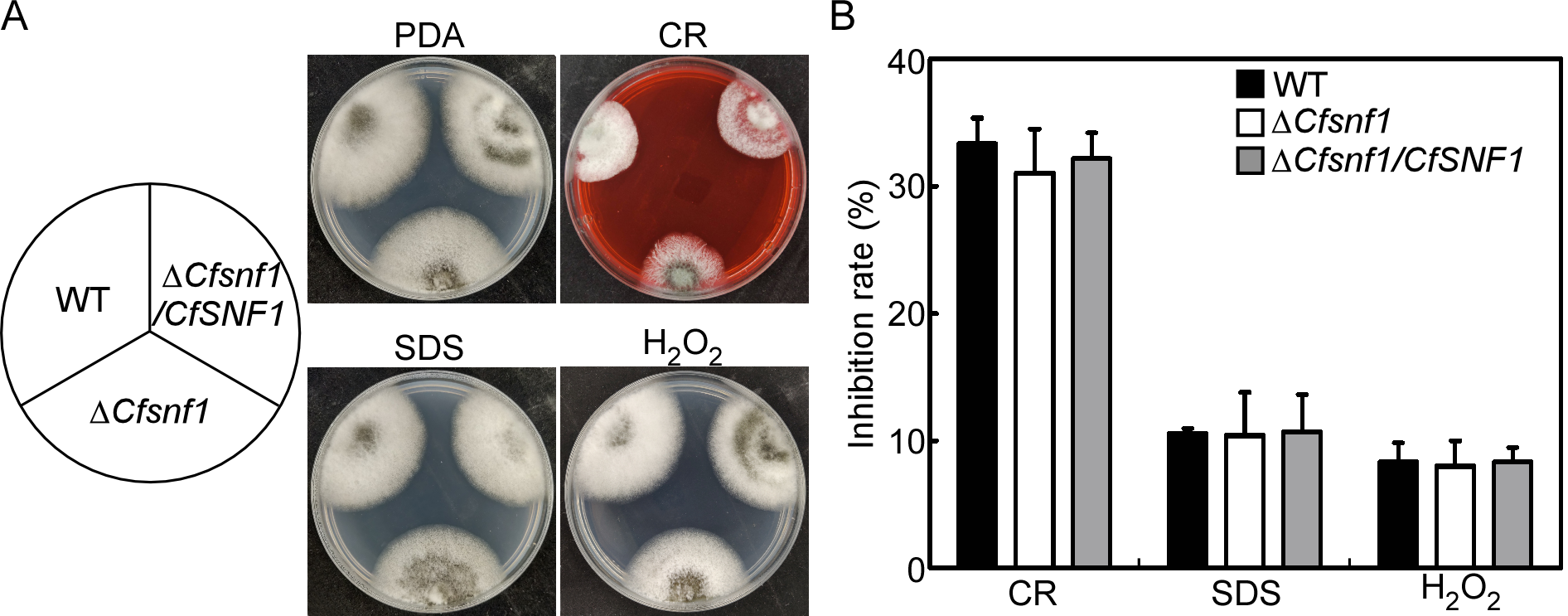

Supplement: Supplementary file 2 — Additional files 2: Figure S2. CfSnf1 is not involved in the tolerance of cell wall stress and oxidative stress. (A) The WT, ΔCfSnf1 and ΔCfSnf1/CfSNF1 strains were cultured onto PDA plates with different cell wall stresses (CR and SDS) and oxidative stress (H2O2) at 28 °C for 4 days and photographed. (B) Statistical analysis of inhibition rates of the strains to cell wall stresses and oxidative stress. Error bars represent SD of three replicates. [file 12863_2019_796_MOESM2_ESM.tif]
