# Supplementary material for: Functional analysis of CfSnf1 in the development and pathogenicity of anthracnose fungus Colletotrichum fructicola on tea-oil tree
Source: BMC Genet. 2019 Dec 5;20:94. doi: 10.1186/s12863-019-0796-y (PMC6896739; doi:10.1186/s12863-019-0796-y)
Supplement: Supplementary file 3 — Additional files 3: Table S1. Primers used in this study. [file 12863_2019_796_MOESM3_ESM.doc]

**Table S1. Primers used in this study**

| **Primer name** | **Sequence (5’-3’)** | **Purpose** |
| --- | --- | --- |
| UF  UR  DF  DR  HYGF  HYGR  NBF  NBR  BWF  HPHR  pYF11F  pYF11R | GGAGTAGTGGTGTATCGAAGGGTC  TTGACCTCCACTAGCTCCAGCCAAGCCGTTGTCGGTCGGGGGGAGGGGGGGC  CAAAGGAATAGAGTAGATGCCGACCGTGATATCTCCTTGACGTTCCGTTAT  CGCTGCCTGTCTCCCTGTAT  GGAGGTCAACACATCAATG  CTCTATTCCTTTGCCCTCG  GCTCACGACCACCTTCAACC  GCTTCCCGCCAATCACTTC  GACGAAGGGGTTGGGTAGTAGGT  GCTGATCTGACCAGTTGC  ACTCACTATAGGGCGAATTGGGTACTCAAATTGGTTAGACTGAGTTCCACCCCGCACAA  CACCACCCCGGTGAACAGCTCCTCGCCCTTGCTCACGTCCGCCTCGGCTAATTGCAT | amplify *CfSNF1* 5’ flank sequence  amplify *CfSNF1* 5’ flank sequence  amplify *CfSNF1* 3’ flank sequence  amplify *CfSNF1* 3’ flank sequence  amplify *HPH* sequence  amplify *HPH* sequence  amplify *CfSNF1* gene sequence  amplify *CfSNF1* gene sequence  validation of *CfSNF1* gene deletion  validation of *CfSNF1* gene deletion  amplify complemented sequence  amplify complemented sequence |
